# Experimental analysis of acupuncture's effects on hyperandrogenemia and related symptoms in PCOS patients

**DOI:** 10.3389/fmed.2025.1531506

**Published:** 2025-07-24

**Authors:** Yu-Xiao Liu, Yan-Hua Han, Yue Jiang, Jiao Zhang, Yue-Hui Zhang

**Affiliations:** ^1^First Clinical Medical College, Heilongjiang University of Chinese Medicine, Harbin, China; ^2^Gynecology I Department, First Affiliated Hospital Heilongjiang University of Chinese Medicine, Harbin, China; ^3^Outpatient Department, Second Affiliated Hospital Heilongjiang University of Chinese Medicine, Harbin, China

**Keywords:** acupuncture, polycystic ovarian syndrome, hyperandrogenemia, treatment, traditional Chinese medicine (TCM)

## Abstract

Traditional Chinese medicine (TCM) has seen a surge in interest in acupuncture as a treatment for polycystic ovary syndrome (PCOS), a condition marked by hyperandrogenism, irregular ovulation, and polycystic ovaries. Acupuncture has been shown to dramatically lower androgen levels in PCOS patients; this effect may be achieved by reducing the secretion of neurotransmitters in the hypothalamic-pituitary-gonadal (HPG) axis and interfering with the hypothalamic-pituitary-adrenal (HPA) axis and its cortisol release. Furthermore, by enhancing glucose metabolism, decreasing inflammation in adipose tissue, and enhancing intercellular communication pathways, acupuncture may indirectly address hyperandrogenemia. Although acupuncture has the potential to treat PCOS, further research is necessary to fully understand its mechanism. It is advised that more clinical research integrating molecular biology and biochemical methods be conducted in the future to determine the precise mechanism of acupuncture's effects on PCOS and to offer new suggestions for managing the condition's symptoms.

## 1 Introduction

Polycystic ovary syndrome (PCOS) is a common gynecological endocrine disorder that significantly affects women's health during their reproductive years. Worldwide, PCOS affects 5%−18% of women, and its prevalence is increasing (1, 2). PCOS is a primary cause of metabolic abnormalities and female infertility. Additionally, a substantial proportion of affected individuals exhibit insulin resistance, obesity, acne, and hirsutism. Moreover, patients with PCOS frequently experience anxiety and face an elevated risk of developing long-term comorbidities such as diabetes and cardiovascular diseases (3). The pathogenesis of PCOS remains incompletely understood and involves multiple contributing factors. Contemporary research indicates that factors, including genetic predisposition (4), gut microbiota dysbiosis (5), oxidative stress (6), and unhealthy lifestyles (7), may contribute to PCOS pathogenesis. In addition to hyperandrogenism, abnormal luteinizing hormone and follicle-stimulating hormone levels are characteristic endocrine manifestations of PCOS (8). Furthermore, adverse emotional states, including stress and anxiety, as well as chronic stress, disrupt normal physiological functions of the hypothalamic-pituitary-gonadal (HPG), and hypothalamic-pituitary-adrenal (HPA) axes (9). Consequently, dysregulation of reproductive endocrine axes (e.g., HPG and HPA) induces hormonal disturbances, thereby contributing to the pathogenesis of PCOS.

As a traditional Chinese medical practice, acupuncture stimulates specific acupoints to modulate physiological processes (10). Acupuncture is thought to alleviate PCOS symptoms by regulating qi and blood circulation, enhancing metabolism, and modulating the neuroendocrine system (11). The “Kidney Qi-Tiankui-Chongren-Baogong” mechanism in traditional Chinese medicine (TCM) corresponds to the hypothalamus-pituitary-ovary-uterus reproductive axis in modern medicine, providing a theoretical foundation for acupuncture treatment of PCOS. Current pharmacological treatments for PCOS, including hormone therapy and metformin, may cause adverse effects such as chest discomfort, headaches, and fatigue (12). In contrast, acupuncture therapy typically exhibits minimal adverse effects, primarily mild local reactions including redness, swelling, and pain (13). It is widely utilized across multiple medical disciplines, including gynecology and internal medicine, particularly in Asian regions (14). Acupuncture has gained significant research attention recently due to its favorable safety profile and minimal adverse effects, making it a popular alternative to pharmacological therapies for PCOS management. Several clinical studies have investigated potential therapeutic benefits of acupuncture for PCOS, including effects on hyperandrogenism, ovulation induction, menstrual cycle regulation, and insulin sensitivity (15). Although preliminary studies suggest potential benefits of acupuncture for PCOS, its precise mechanisms remain unclear, warranting further investigation to confirm efficacy and elucidate mechanisms of action (16).

This review aims to provide a novel perspective on acupuncture's role in PCOS management by examining its effects on hyperandrogenism and associated endocrine mechanisms. Through systematic analysis of current experimental evidence, we will evaluate the clinical efficacy of acupuncture and identify endocrine-related mechanistic changes. This synthesis will provide substantiated evidence for clinical translation and advance a comprehensive understanding of acupuncture's therapeutic potential in PCOS.

## 2 Acupuncture treatment for hyperandrogenism in PCOS patients

Clinical studies investigating acupuncture's effects on hyperandrogenism in PCOS have demonstrated positive outcomes. Clinical evidence indicates that PCOS patients undergoing acupuncture therapy exhibit significant reductions in serum testosterone levels following treatment. For instance, Jedel et al. (17) reported that 16 weeks of low-frequency electroacupuncture (EA) significantly reduced testosterone levels and increased estradiol levels in PCOS patients. Furthermore, low-frequency electroacupuncture demonstrated superior efficacy in ameliorating hyperandrogenism compared to exercise interventions. Compared with the exercise group, the low-frequency electroacupuncture (EA) group exhibited a 25% reduction in circulating testosterone (T), along with 30 and 28% decreases in androsterone glucuronide and 3α-androstanediol levels, respectively. Menstrual frequency significantly increased from 0.28 episodes at baseline to 0.69 episodes per month (*p* < 0.05). The EA intervention induced a 19% decline in dihydrotestosterone (DHT) levels, demonstrating superior efficacy compared to the 2% reduction observed in the exercise group (*p* < 0.01). Concurrently, acne severity scores showed a 32% improvement in the EA group, with concomitant reductions in estrogen levels: estrone (E1, 19%), estrone sulfate (E1-S, 12%), and estradiol (E2, 3%; all *p* < 0.05). Similarly, a seminal study by Stener-Victorin et al. (18) demonstrated that EA intervention significantly reduced circulating testosterone (22% reduction) and dihydrotestosterone (DHT, 12% decrease) in obese PCOS patients. Concomitantly, adipose tissue analysis revealed an 18% decline in testosterone concentration along with a 13% reduction in androstenedione levels compared to baseline measurements. A randomized controlled trial (RCT) involving PCOS patients revealed that the Dong's acupuncture (DA) intervention group achieved a more pronounced reduction in total testosterone levels compared with the pharmaceutical group receiving cyproterone acetate/ethinylestradiol (CPA/EE). Quantitative analysis demonstrated significantly greater decreases in the DA group (−0.45 nmol/L, 95% CI −0.52 to −0.38) vs. the CPA/EE group (−0.17 nmol/L, 95% CI −0.24 to −0.10), with the difference between groups reaching statistical significance (*p* = 0.008) (19). Although limited by a modest sample size (*n* = 60), the trial demonstrated clinically meaningful superiority of acupuncture therapy over conventional pharmacotherapy in lowering androgen levels. Notably, between-group differences exceeded the minimum clinically important differences for testosterone parameters (Δ = 0.28 nmol/L, ES = 0.62). Concurrently, preliminary evidence from case reports suggested that auricular acupuncture (AA) may ameliorate hyperandrogenemia in infertility management. Of particular mechanistic interest, one observational study (non-PCOS cohort) reported 18%−22% reductions in serum androgens following AA intervention (95% CI −25% to −15%, *p* = 0.032), though confirmation through powered RCTs remains warranted (20). Clinical randomized controlled trials demonstrate that auricular electroacupuncture effectively ameliorates hyperandrogenism and other PCOS symptoms as adjuvant therapy (21). Furthermore, integrative therapies combining acupuncture with segmental moxibustion and herbal formulations based on TCM syndrome differentiation have significantly reduced hyperandrogenism in PCOS patients with kidney deficiency and phlegm-dampness constitutions (22). Collectively, these findings indicate that acupuncture significantly improves hyperandrogenism in PCOS. Notably, investigators reported concomitant reductions in other PCOS-related symptoms. Park et al. (23) demonstrated that acupuncture intervention significantly improved menstrual cyclicity in PCOS patients. Li et al. (24) reported that electroacupuncture increased ovulation rates and improved live birth rates in PCOS patients following ovulation induction. Additionally, multiple studies document improvements in metabolic parameters, reduced abdominal adiposity, decreased acne severity, and weight reduction (25–27). These clinical findings indicate that acupuncture not only alleviates multiple PCOS-related symptoms but also significantly modulates androgen levels.

Acupuncture therapy for PCOS demonstrates a favorable safety profile, with minimal risk of serious adverse events. Clinical trial participants typically report only mild transient reactions, primarily localized and temporary erythema or bruising at needle insertion sites. These feelings of discomfort are typically mild and transient. As a result, acupuncture is thought to be a rather safe therapeutic choice for PCOS sufferers (28). The selection of acupuncture sites was predominantly concentrated in the lumbosacral region [e.g., Qihai (CV6), Guanyuan (CV4)], lower extremities [e.g., Sanyinjiao (SP6), Yinlingquan (SP9)], and hands (e.g., Dong's acupuncture). Specialized acupuncture was primarily focused on the auricular region (e.g., auricular seed application). These determinations were consistently made based on the practitioner's diagnostic approach and the patient's symptomatic presentation, with no standardized protocols established to date.

## 3 Acupuncture improves PCOS through the reproductive endocrine axis

### 3.1 Acupuncture improves PCOS by modulating GnRH pulsatility within the HPG axis

The GnRH pulse generator, located in the arcuate nucleus, is regulated by the interplay of kisspeptin/neurokinin B/dynorphin (KNDy) neurons and is finely tuned by steroid feedback (progesterone, estrogen) and metabolic signals such as insulin and leptin (29, 30). In PCOS, reduced progesterone negative feedback combined with elevated ovarian androgen levels accelerates GnRH pulse frequency, leading to excessive LH secretion, an increased LH/FSH ratio, and consequent ovarian hyperandrogenism (31–33).

Li et al. (34) demonstrated that the neuropeptide Y (NPY) mRNA level in the hypothalamus of adolescent PCOS rats was increased, and electroacupuncture could reverse the NPY level, while the estrous cycle disorder and ovarian morphological abnormalities were significantly improved. Mechanistically, acupuncture downregulates hypothalamic neuropeptide Y while upregulates β-endorphin expression in the arcuate nucleus, thereby stabilizing KNDy neuronal activity and reducing GnRH pulse frequency (35). These neuroendocrine adjustments re-establish a balanced LH/FSH ratio, attenuate ovarian androgen synthesis, and ultimately improve menstrual regularity and ovarian function in PCOS patients (36). In PCOS rat models, acupuncture normalizes the ovarian neuroendocrine milieu by down-regulating nerve growth factor (NGF) and endothelin-1 (ET-1), key mediators of aberrant follicular development and vascular dysregulation, thereby improving folliculogenesis (37). Concurrently, electroacupuncture modulates hypothalamic regulators of GnRH pulsatility by suppressing arcuate-nucleus neuropeptide Y and NGF expression and reducing GnRH mRNA transcription to stabilize KNDy neuronal activity and correct the LH/FSH ratio (38). By rebalancing the HPG axis, acupuncture also mitigates superovulation-induced ovarian hyperstimulation syndrome, as demonstrated by reduced serum estradiol, increased pituitary estrogen receptor β expression, and higher counts of healthy antral and mature follicles (39).

In conclusion, current evidence demonstrates that acupuncture modulates the release of neuroendocrine factors, such as NGF, NPY, and ET-1, providing mechanistic insights and future research avenues in treating hyperandrogenism in PCOS.

### 3.2 Acupuncture improves PCOS by affecting the hypothalamic-pituitary-adrenal axis

Controlling stress responses and maintaining internal metabolic balance are the responsibilities of the HPA axis, an essential physiological system. The HPA axis triggers the release of adrenal cortex hormones, mainly cortisol, in response to stress or danger. These hormones help the body release stored energy, improve alertness, and adjust to stressful situations. The immune system and the HPA axis are intimately related; cortisol can regulate immunological balance by reducing inflammation and immune cell activity. Overall, the HPA axis is a critical physiological system responsible for regulating stress responses and maintaining internal metabolic homeostasis. In response to stress or perceived threats, the HPA axis initiates the release of adrenal cortex hormones, primarily cortisol. These hormones facilitate the mobilization of stored energy, enhance alertness, and support the body's adaptation to stressful conditions. The immune system and the HPA axis are closely interconnected; cortisol plays a key role in modulating immune homeostasis by suppressing inflammation and regulating immune cell activity. In pathological conditions, decreased melatonin secretion and new corticotropin-releasing hormone receptor gene mutations (CRHR1 and CRHR2) lead to overactivation of the HPA axis, leading to the risk of developing PCOS (40, 41). Stressful stimuli resulted in elevated salivary cortisol and cortisone levels in PCOS patients, which were positively correlated with circulating androstenedione and DHEA. At the same time, higher HPA-axis activity correlates with higher adrenal androgen output and a worse metabolic profile, circulating androstenedione, salivary cortisol (SalF), and cortisone (SalE) (42). According to previous studies, dysregulation of the HPA axis in patients with PCOS may lead to elevated androgen levels and chronic inflammation (43). Furthermore, HPA axis dysfunction has been implicated in other clinical manifestations of PCOS, such as obesity and hirsutism (42, 44). Increasing attention is being directed toward the role of acupuncture in modulating HPA axis activity, and several mechanisms have been proposed. Evidence suggests that electroacupuncture may alleviate depressive-like behaviors and suppress excessive HPA axis activation in rats subjected to chronic unpredictable mild stress (45). Clinical studies have also demonstrated that acupuncture can significantly reduce generalized anxiety disorder in perimenopausal women by modulating the HPA axis and inhibiting cortisol release (46). Similar findings have been reported in patients with allergic rhinitis and post-stroke anxiety (47, 48). Given the high prevalence of anxiety and depression in patients with PCOS, these findings suggest that electroacupuncture may improve hyperandrogenism by attenuating HPA axis hyperactivity.

**Figure 1:**
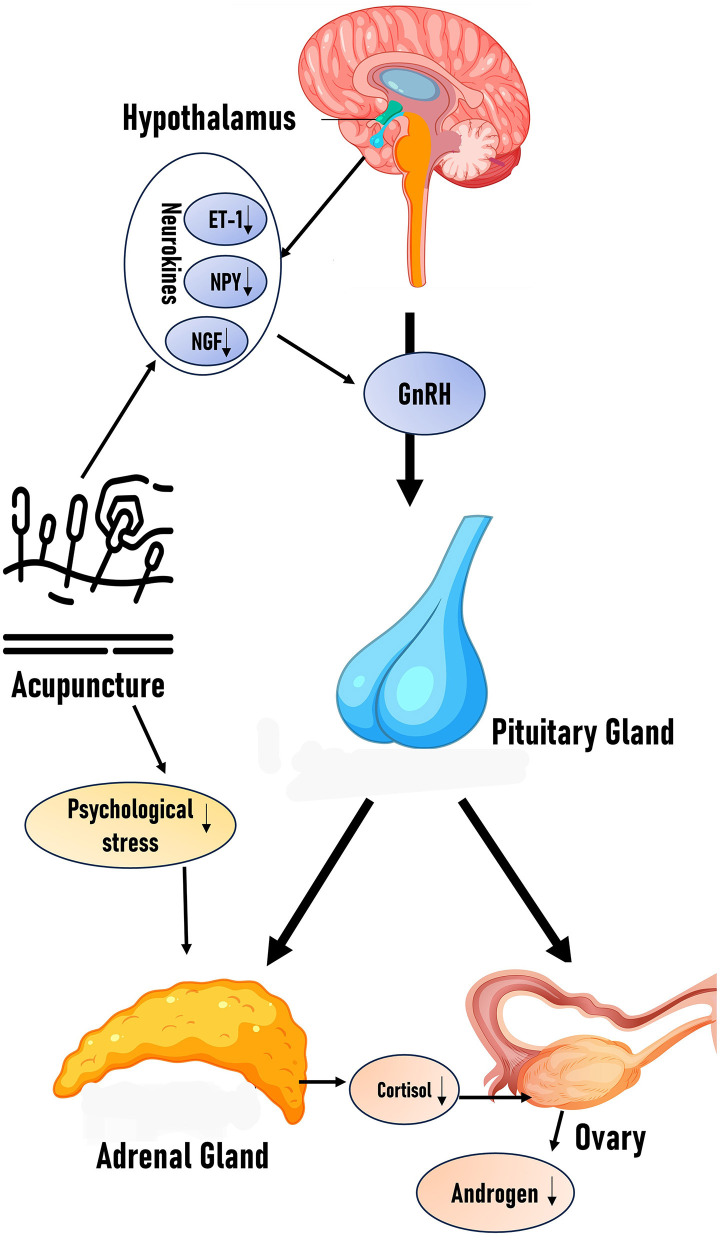
Effects of acupuncture on HPG axis and HPA axis: the figure schematically illustrates how acupuncture modulates the balance of the HPA and HPG axes by reducing neurotransmitters (ET-1, NPY, and NGF) and stressors, along with the proposed hypothesis.

In PCOS, HPA axis hyperactivity frequently disrupts endocrine homeostasis. Acupuncture may restore hormonal balance by attenuating HPA axis hyperactivity. We propose that acupuncture ameliorates PCOS symptoms through HPA axis modulation, subsequently regulating neuroendocrine-immune crosstalk. Currently, direct experimental evidence linking acupuncture-mediated HPA axis modulation to hyperandrogenism amelioration remains limited. Future clinical and preclinical studies investigating this pathway would provide stronger mechanistic validation for acupuncture's therapeutic effects (Figure 1).

## 4 Acupuncture improves PCOS by intervening in cellular signaling pathways

### 4.1 Hypothesis on the mechanisms by which electroacupuncture modulates ovarian-related signaling pathways

To elucidate PCOS pathophysiology and treatment mechanisms, recent research has increasingly focused on signaling molecules, expanding mechanistic investigations to non-pharmacological therapies. Accumulating evidence implicates multiple signaling pathways that are involved in acupuncture's therapeutic actions for PCOS. Primarily, electroacupuncture modulates ovarian signaling pathways to restore ovulatory function in anovulatory PCOS patients. In ovarian granulosa cells, electroacupuncture downregulates chromatin-interacting long non-coding RNA (LncMEG3), PI3K/AKT/mTOR pathway components, and autophagy markers (SQSTM1/p62 and LC3II/LC3I ratio). Conversely, it upregulates ovulation-promoting hormone receptors. These findings indicate that electroacupuncture suppresses the PI3K/AKT/mTOR pathway and attenuates granulosa cell autophagy via LncMEG3 downregulation, thereby promoting ovulation (49). Given that dysregulated granulosa cell autophagy elevates ovarian androgen production (50), we propose that electroacupuncture ameliorates hyperandrogenism through autophagy suppression.

### 4.2 Hypothesis on the mechanisms by which electroacupuncture modulates signaling pathways related to glucose and lipid metabolism

Furthermore, electroacupuncture stimulation can treat insulin resistance in PCOS by modifying insulin receptor substrate (IRS) signaling pathways and regulating glucose and lipid metabolism. Specifically, Xiang et al. (51) demonstrated that electroacupuncture boosts IRS-1 expression in PCOS patients, thereby activating the PI3K/GLUT4 signaling pathway and enhancing insulin signal transduction effectiveness. This improvement in insulin signaling is significant because hyperinsulinemia resulting from insulin resistance is known to enhance ovarian androgen synthesis, a process linked to diminished insulin signaling pathway efficiency (52). Complementing these clinical findings, several animal studies have explored the molecular mechanisms of acupuncture for PCOS from various perspectives. For instance, acupuncture was reported to activate the PI3K/AKT pathway (closely related to the PI3K pathway involved in insulin signaling), enhancing endometrial angiogenesis in PCOS rats. This effect increased the number of implantation sites and improved the rats' reproductive potential (53). This is particularly relevant since hyperandrogenemia, a hallmark of PCOS, often exacerbated by insulin resistance, increases miscarriage risk by impairing endometrial receptivity (54).

### 4.3 Hypothesis on the mechanisms and gene expression changes induced by electroacupuncture intervention

Furthermore, studies have investigated potential miRNA-mRNA co-expression networks in diabetes and PCOS. Specifically, they discovered that acupuncture significantly increases PLA2G4A expression and decreases miR-32-3p levels. Critically, subsequent research demonstrated that miR-32-3p downregulates PLA2G4A (phospholipase A2, group IVA) expression, and this interaction impacts glucose metabolism in PCOS patients (55). The significance of PLA2G4A dysregulation extends beyond metabolism, as aberrant expression of this gene in PCOS patients' oocytes is also linked to sex hormone receptors (56), highlighting its broader role in PCOS pathophysiology. This illustrates how acupuncture can affect the genetic level by modulating specific gene expression, such as through the miR-32-3p/PLA2G4A axis. Beyond miRNA-mediated mechanisms, acupuncture also directly influences tissue-specific gene expression related to metabolic dysfunction. For instance, manual acupuncture improved glucose clearance and primarily modified gene expression in visceral fat tissue. Experiments by Johansson et al. (57) further demonstrated this in a PCOS rat model: electroacupuncture countered DHT-induced changes by altering gene expression (decreased Tbc1d1 in soleus, increased Nr4a3 in mesenteric fat) and protein expression (increased pAS160/AS160, Nr4a3; decreased GLUT4). These findings on acupuncture's impact on fat tissue gene/protein expression are particularly relevant given clinical observations: studies in women with PCOS show that serum testosterone levels positively correlate with subcutaneous and visceral fat thickness as well as adipocyte size (58), suggesting a link between androgen excess and adipose tissue dysfunction that acupuncture may help modulate.

**Figure 2:**
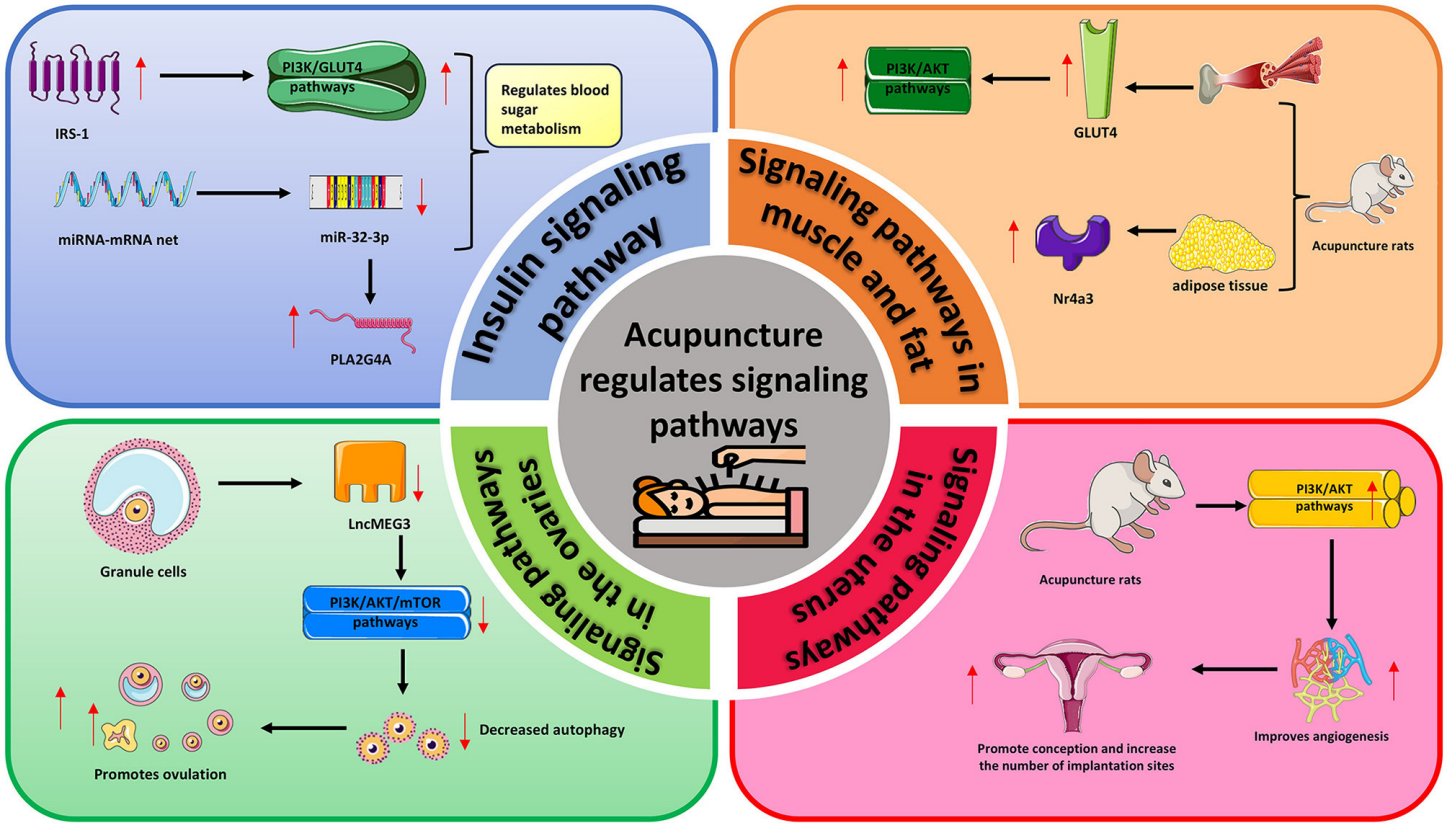
Acupuncture treats PCOS through multiple signaling pathways.

In conclusion, acupuncture may provide therapeutic effects in PCOS by modulating various signaling pathways involved in adipose tissue, ovarian function, skeletal muscle, the uterus, and insulin regulation. It may also influence gene expression. Despite these promising findings, few studies have specifically investigated the direct mechanisms by which acupuncture alleviates hyperandrogenism. Moreover, further research is needed to elucidate the specific molecular pathways involved and the interactions among these pathways. Currently, little is known about how acupuncture affects molecular expression at the genetic level in patients with PCOS. Continued investigation into the genetic and molecular mechanisms may yield more precise insights and inform the development of targeted acupuncture-based therapies (Figure 2).

## 5 Acupuncture treats hyperandrogenism by improving blood sugar metabolism in PCOS patients

### 5.1 How does acupuncture improve glucose metabolism in PCOS

Insulin resistance and glycemic dysregulation are strongly associated with PCOS, leading to elevated circulating insulin levels. Consequently, patients with PCOS have an increased risk of developing diabetes. Studies have shown that glucose intolerance is a common clinical manifestation in women with PCOS (59). It is well-established that insulin primarily promotes androgen production in the ovary, contributing to hyperandrogenism, a hallmark feature of PCOS. However, elevated androgen levels also contribute to the development of insulin resistance, perpetuating a vicious cycle of hyperandrogenism, compensatory hyperinsulinemia, and insulin resistance, ultimately leading to the chronic progression of PCOS (60). Although the precise etiologies of hyperinsulinemia and hyperandrogenemia remain unclear, addressing either condition is essential for the management of metabolic disorders. Numerous studies have demonstrated that acupuncture can modulate glucose metabolism through multiple signaling pathways. Therefore, investigating the effects of acupuncture on blood glucose regulation may help establish its utility in treating hyperglycemia and improving insulin sensitivity in patients with PCOS. Relevant animal studies suggest that electroacupuncture may alleviate insulin resistance in PCOS by modulating insulin signaling pathways. This effect is mediated by upregulating GLUT4 expression in skeletal muscle and activating the PI3K/AKT signaling pathway in rats (61). Hence, electroacupuncture at ST25 reduced homeostasis model assessment of insulin resistance and hemoglobin A1c levels in diabetic model rats. Concurrently, electroacupuncture at ST25 reversed the reductions in adiponectin (Adipo) and AdipoR1 levels in skeletal muscle induced by insulin resistance. Compared with the model group, electroacupuncture further controlled obesity- and inflammation-associated factors [e.g., reduced leptin, tumor necrosis factor-α (TNF-α), and interleukin-1β (IL-1β)]. Relative to the non-EA group, EA reversed alterations in hippocampal mRNA levels of IRS-1, IRS-2, PI3K, Akt, insulin-degrading enzyme, glycogen synthase kinase 3 alpha (GSK3α), and glycogen synthase kinase 3 beta (GSK3β), with higher Akt levels observed in the EA group (62–64). Further studies have demonstrated that acupuncture can significantly improve various PCOS-related symptoms in mice, reduce insulin resistance, and normalize fasting blood glucose levels (65). Clinical studies have shown that individuals with type 2 diabetes experience significant reductions in both average stress scores and fasting blood glucose levels following acupuncture treatment (66). Concurrently, Xu et al. (62) reported that electroacupuncture improves both the morphology and function of pancreatic β-cells in T2DM rats, thereby enhancing overall pancreatic function. These effects are attributed to electroacupuncture-induced suppression of inflammation and modulation of the pancreatic intrinsic nervous system via upregulation of neuropeptide Y and choline acetyltransferase expression.

### 5.2 Hypothesis on the improvement of hyperandrogenism through acupuncture-mediated modulation of glucose metabolism

In patients with PCOS, elevated blood glucose levels trigger a compensatory increase in insulin secretion due to underlying insulin resistance. This hyperinsulinemia, in turn, contributes to excessive androgen production in the ovaries (67). Furthermore, hyperglycemia may influence androgen levels via metabolic pathways by downregulating insulin-sensitive glucose transporter protein (GLUT4), thereby reducing glucose uptake in ovarian cells (68). Elevated insulin levels can increase IGF-1 expression, which subsequently enhances androgen synthesis in the ovaries, while hyperglycemia may concurrently activate AMPK, further affecting androgen production (69). Emerging evidence suggests that acupuncture may modulate IGF-1 expression (70). This means that by activating particular acupoints (ST29 and SP6 acupuncture points), acupuncture may improve insulin sensitivity and improve cells' ability to absorb glucose. As a result, insulin levels drop, which in turn lessens the signals that cause the ovaries to overproduce androgens. This can be achieved by activating the AMPK pathway since AMPK promotes intracellular glucose metabolism (16). When hyperglycemic, reactive oxygen species (ROS) can rise dramatically. ROS have the ability to directly disrupt the ovaries' androgen synthesis pathways, which can impact the generation of androgens. Based on this study, acupuncture is hypothesized to attenuate oxidative stress by reducing reactive oxygen species (ROS) generation, thereby leading to decreased testosterone levels in PCOS patients, though no experimental evidence supports this mechanism (71). Elevated blood glucose levels can activate nuclear factor-kappa B (NF-κB), thereby promoting the synthesis of pro-inflammatory cytokines. These inflammatory mediators may directly or indirectly impair testosterone synthesis in the ovaries, thereby exacerbating the symptoms of PCOS (72). The anti-inflammatory effects of acupuncture may be mediated by inhibition of the NF-κB signaling pathway, resulting in reduced androgen levels through suppression of inflammatory protein synthesis (73).

In conclusion, studies have suggested that acupuncture, a traditional Chinese medical practice, may offer therapeutic benefits in regulating glucose metabolism. Acupuncture may improve glycemic control through multiple mechanisms, including modulation of insulin sensitivity and secretion, anti-inflammatory effects, and central nervous system regulation. Given the triad of hyperinsulinemia, hyperandrogenemia, and insulin resistance, it is hypothesized that acupuncture may indirectly reduce androgen levels in patients with PCOS by improving pancreatic islet function and glucose metabolism. Additionally, acupuncture has been shown to significantly improve glycemic control in patients with PCOS and may contribute to improved quality of life (74). As research progresses, acupuncture may emerge as an effective adjunctive therapy for managing blood glucose levels in patients with PCOS.

## 6 PCOS is ameliorated through acupuncture-mediated improvements in adipose tissue functionality and related metabolic parameters

### 6.1 How does acupuncture improve adipose tissue metabolism in PCOS

Inflammation of adipose tissue is closely associated with PCOS. Beyond serving as an energy storage site, adipose tissue functions as an active endocrine organ, secreting various pro-inflammatory cytokines such as interleukin-6 (IL-6), tumor necrosis factor-α (TNF-α), and others (75). In patients with PCOS, adipose tissue inflammation is often markedly elevated, exacerbating clinical symptoms and contributing to abnormal follicular development, hyperandrogenism, and related complications (76). Acupuncture is believed to reduce adipose tissue inflammation, potentially alleviating endocrine disturbances in patients with PCOS. Studies have shown that electroacupuncture can modulate adipose tissue inflammation in obese rats by regulating catecholamine signaling, inhibiting the NLRP3 inflammasome pathway, and ultimately reducing inflammatory responses (77). Researchers suggest that electroacupuncture may mitigate obesity-related inflammation through these mechanisms. Furthermore, by modulating adipose tissue inflammation, electroacupuncture may contribute to the amelioration of hyperandrogenemia. Women with PCOS are at increased risk of developing non-alcoholic fatty liver disease (NAFLD), as elevated androgen levels can induce hepatic steatosis (78). Concurrently, electroacupuncture has demonstrated significant therapeutic effects in rat models of NAFLD characterized by hepatic inflammation. Therefore, by reducing hepatic inflammation, electroacupuncture may also attenuate adipose tissue inflammation and alleviate symptoms of NAFLD (79). Furthermore, the study found that electroacupuncture at Zhongwan (CV12) and Guanyuan (CV4) significantly reduced adiponectin and TNF-α levels in 12–14-week-old obese rats, while increasing leptin levels and significantly decreasing the leptin-to-adiponectin ratio in 3-week-old obese rats (80). Simultaneously, research has demonstrated that intraovarian lipid exposure and the ensuing inflammation and lipotoxicity may be factors in women's elevated testosterone production (81). Therefore, through altering inflammatory lipid metabolism, electroacupuncture may be used to treat hyperandrogenemia. According to Zhang et al's study (82), EA normalized serum dihydrotestosterone (DHT) and progesterone levels in PCOS model rats. Tenericutes at the phylum level and Prevotella_9 at the genus level, which were closely associated with body weight, exhibited significant alterations in the PCOS group before and after EA treatment. Consequently, reductions in visceral and subcutaneous fat content were achieved in PCOS rats. Moreover, an inverse correlation is observed between total testosterone levels and gut microbiota diversity in PCOS patients, while reduced gut microbiota diversity is consistently associated with obesity (83). In addition to modulating the gut microbiota, electroacupuncture may also reduce testosterone levels. Furthermore, acupuncture may improve the metabolic status of obese individuals with PCOS by regulating pathways associated with glucose metabolism (84). These studies offer novel insights into potential therapeutic strategies for managing obesity in PCOS. A randomized controlled clinical trial demonstrated that acupuncture can alter gene expression in adipose tissue of obese women, influencing biological processes including lipid metabolism, olfactory transduction, and gamma-aminobutyric acid signaling pathways (85). This provides important insights into the underlying mechanisms of acupuncture therapy. Multiple studies have proposed diverse mechanisms through which acupuncture regulates fat metabolism. Among these findings, electroacupuncture has been shown to activate the PI3K/Pten/Thbs1 signaling pathway, thereby promoting adipose tissue browning in obese mice. This suggests that electroacupuncture may help alleviate obesity by promoting angiogenesis and adipose tissue browning (86). Alternatively, electroacupuncture may enhance adipose tissue browning through Sirtuin-1–dependent mitochondrial biogenesis and PPARγ deacetylation (87). In addition, individualized acupuncture treatment protocols tailored to patient-specific characteristics and obesity subtypes may be developed.

### 6.2 Hypothesis on the improvement of hyperandrogenism through acupuncture-mediated regulation of adipose tissue metabolism

**Figure 3:**
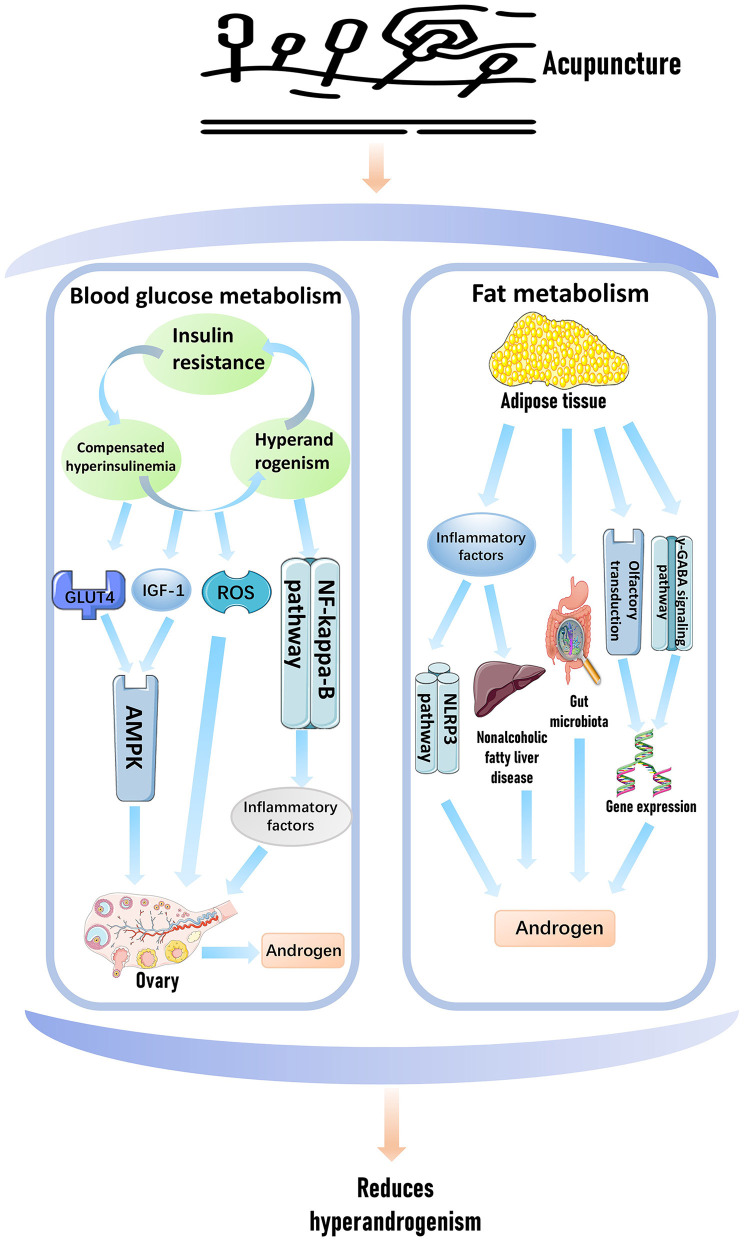
Acupuncture treats hyperandrogenism by intervening in blood sugar and fat metabolism.

Elevated testosterone levels in patients with PCOS are closely associated with impaired lipid metabolism. In patients with PCOS, elevated cholesterol levels may provide increased substrate availability for androgen synthesis, particularly for androgenic steroid hormones, thereby contributing to elevated androgen levels (88). Acupuncture may reduce low-density lipoprotein and total cholesterol levels, thereby decreasing the availability of cholesterol precursors required for testosterone synthesis (89). This effect is likely mediated through the regulation of 3β-hydroxysteroid dehydrogenase, a key enzyme in the androgen biosynthesis pathway (90). Inflammatory conditions in adipose tissue are frequently observed in patients with PCOS. Chronic inflammation can lead to the release of various cytokines from adipose tissue, which may disrupt ovarian androgen synthesis pathways and increase androgen levels (91). By reducing adipose tissue inflammation, acupuncture may help regulate testosterone levels. It may suppress the release of inflammatory mediators such as IL-6 and TNF-α from adipose tissue (92). These cytokines are known to influence androgen levels in patients with PCOS (93). Additionally, acupuncture may modulate signaling pathways such as NLRP3, PI3K, and AMPK, thereby improving obesity-related parameters and subsequently reducing testosterone levels (77, 86, 94) (Figure 3).

However, it is important to note that experimental evidence supporting the use of acupuncture for treating hyperandrogenemia remains limited, particularly regarding its effects on the central nervous system, peripheral metabolism, and genetic mechanisms. Although several animal and clinical studies have demonstrated the efficacy of acupuncture in treating PCOS, the quality of these studies is inconsistent, and the underreporting of negative outcomes may introduce bias. Moreover, there is a continued lack of large-scale systematic reviews and meta-analyses to synthesize and critically assess the current body of research. Therefore, future research should focus on increasing clinical sample sizes and supplementing findings with relevant animal studies, which may yield more robust and reliable outcomes.

## 7 Acupuncture treatment and quality of life in patients with PCOS

It is well-established that individuals with PCOS frequently experience emotional symptoms, including a diminished health-related quality of life. Among these emotional disturbances, anxiety and depression are particularly prominent. In TCM, liver qi stagnation is commonly considered the underlying cause of anxiety and depression. Consequently, therapeutic strategies focus on alleviating depression and soothing the liver. Acupuncture therapy often involves needle insertion at acupoints along or near the liver meridian. Chang et al. (95) initially demonstrated in a randomized clinical trial that acupuncture had beneficial effects on emotional well being and lipid metabolism in PCOS patients. The present study found that after a 4-month acupuncture intervention, patients exhibited improved lipid metabolism and reduced symptoms of anxiety and depression. These findings support the use of acupuncture as an adjunctive therapy for individuals with PCOS. Another randomized controlled trial investigated the effects of acupuncture on emotional symptoms and quality of life in PCOS patients. Results indicated that the acupuncture group experienced significant improvements in quality of life scores and a marked reduction in anxiety and depression symptoms, further supporting acupuncture as an effective intervention for enhancing psychological well being in PCOS patients (96). Moreover, studies suggest that both standardized and individualized acupuncture treatments significantly improve the quality of life in PCOS patients. Meta-analytic findings highlight the benefits of moxibustion and traditional Chinese medicine in improving the quality of life and alleviating anxiety and depression symptoms in PCOS patients, particularly in the context of fertility-related concerns (97, 98). Finally, researchers observed that acupuncture reduced symptoms of anxiety and depression in PCOS patients. Concurrently, the SF-36 survey revealed improvements in physical functioning, vitality, general health perception, and overall mental health scores. According to this study, acupuncture may alleviate emotional disturbances in PCOS patients, particularly improving physical functioning, vitality, and mental health (99).

Irregular menstruation, acne, and excessive hair growth are common accompanying symptoms in patients with PCOS. An increasing number of studies have shown that acupuncture may be effective in treating hirsutism, acne, and menstrual irregularities in PCOS patients. Zhou et al. (100) investigated the effects of acupuncture on menstrual frequency in patients with PCOS. The study supported acupuncture as an intervention strategy for improving menstrual frequency in PCOS patients, as a significant increase was observed in the true acupuncture group. Another clinical trial (101) confirmed that electroacupuncture significantly improved hirsutism and reproductive function in PCOS patients. Additionally, in obese PCOS patients, abdominal acupuncture has shown significant benefits in improving menstrual frequency, body mass index, and waist-to-hip ratio (102). Furthermore, a randomized controlled trial on auricular acupressure for PCOS patients demonstrated improvement in acne symptoms in the treatment group (27). Moreover, studies investigating the effects of low-frequency electroacupuncture combined with physical activity in PCOS patients have shown significant improvements in menstrual regularity, acne, and hirsutism (17).

In summary, acupuncture is a complementary and alternative therapy that can improve menstrual regularity, reduce hirsutism and acne, enhance quality of life, and support emotional well being and life satisfaction in patients with PCOS. However, there are certain limitations associated with acupuncture. Notably, acupuncture often requires a prolonged course of treatment, which may be challenging for some patients to adhere to. Furthermore, due to individual variability, the effectiveness of acupuncture can vary significantly, resulting in heterogeneous responses among patients. Therefore, more personalized and tailored treatment approaches are warranted. Moreover, many existing studies have limited follow-up durations and lack a comprehensive analysis of long-term treatment outcomes. Encouragingly, most PCOS patients view acupuncture therapy favorably, particularly those seeking to avoid pharmacological treatments and their associated side effects. Many patients consider acupuncture to be a gentle and natural form of therapy.

## 8 Conclusion

As a safe and cost-effective adjunctive therapy, acupuncture can be integrated with standard pharmacological treatments and lifestyle modifications in PCOS management. While direct evidence from clinical trials or preclinical models demonstrating acupuncture-mediated reduction of circulating androgens remains limited, current clinical and preclinical evidence indicates that acupuncture modulates HPG and HPA axis functions, enhances insulin sensitivity, and attenuates chronic adipose tissue inflammation. These multimodal effects ultimately restore regular menstrual cyclicity, ameliorate hyperandrogenism, and improve psychological well being. Collectively, this evidence supports acupuncture's potential to ameliorate hyperandrogenism.

Mechanistically, acupuncture modulates key pathways regulating steroidogenesis, glucose metabolism, and immune function. Preclinical and clinical studies demonstrate its neuroendocrine regulatory actions through bidirectional modulation of neurotransmitters and hormones. Emerging evidence further indicates that acupuncture alters inflammatory cytokine profiles in adipose tissue, consequently improving metabolic indices.

Future development should focus on personalized acupuncture protocols guided by genetic polymorphisms and validated biomarkers. The identification of reliable biomarkers, including neuroendocrine sensitivity-associated single-nucleotide variants and serum cytokine profiles (103, 104), will enable precise selection of acupoints (e.g., Sanyinjiao [SP6], Qihai [CV6], Guanyuan [CV4], and Zigong [EX-CA1]), optimization of needling parameters, and patient-specific treatment scheduling. Concurrently, rigorously designed multicenter trials with standardized protocols are needed to evaluate long-term safety and therapeutic sustainability.

**Figure 4:**
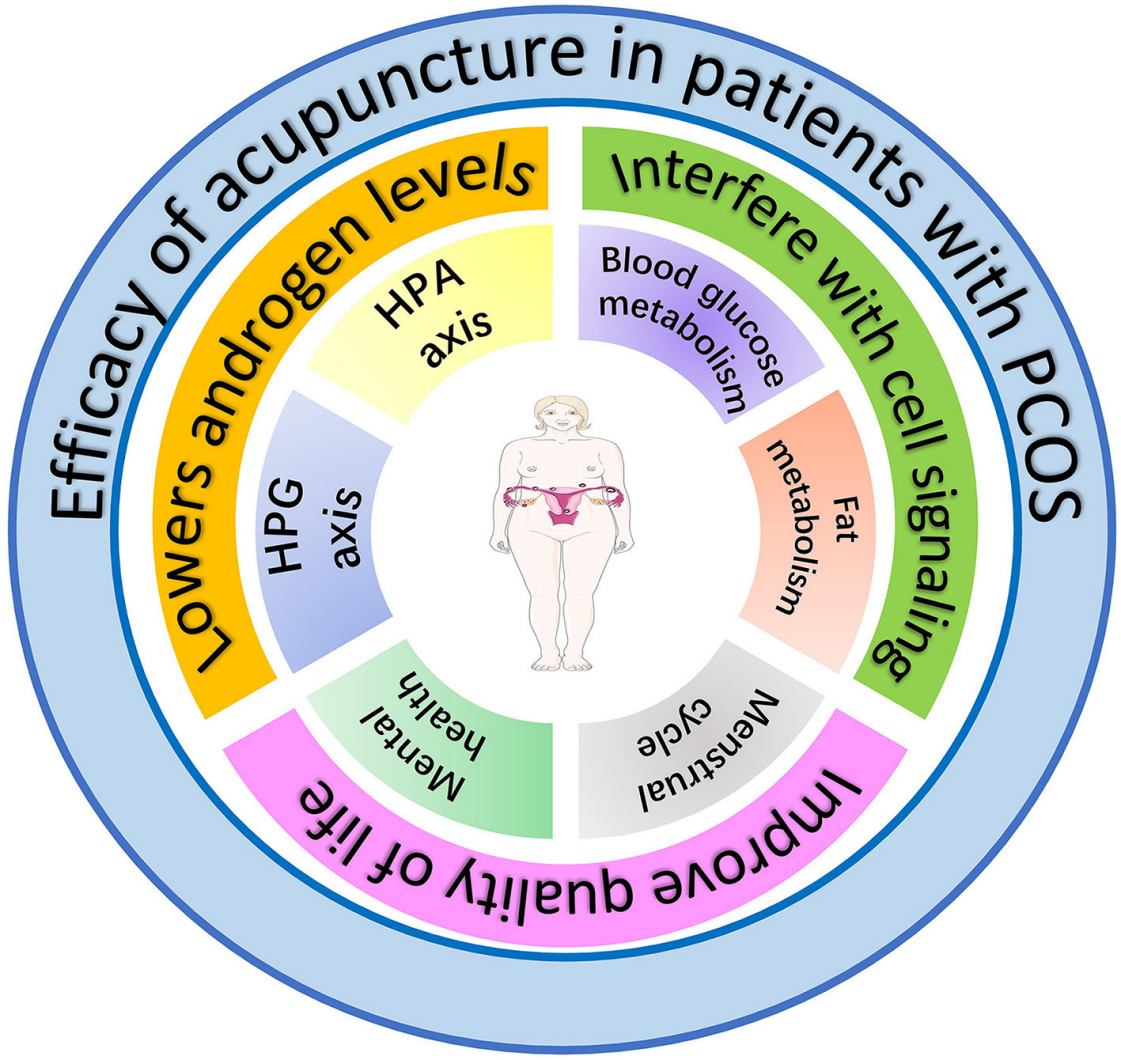
The effects of acupuncture on women with PCOS. The figure hypothesizes that amelioration of PCOS symptoms by acupuncture is achieved through three primary mechanisms: (1) reduction of androgen levels via modulation of the hypothalamic-pituitary-adrenal (HPA) and hypothalamic-pituitary-gonadal (HPG) axes, (2) improvement of glucose and lipid metabolism through regulation of intercellular signaling pathways, and (3) enhancement of quality of life by ameliorating emotional disturbances and menstrual cyclicity.

Establishing internationally standardized guidelines for acupuncture diagnosis, point prescription, and practitioner certification is essential for reproducibility and global implementation. As acupuncture becomes integrated into comprehensive PCOS care pathways, it is poised to evolve as an evidence-based component of personalized medicine, optimizing physiological outcomes and quality of life (Figure 4).
